# *Onthophagus pilauco* sp. nov. (Coleoptera, Scarabaeidae): evidence of beetle extinction in the Pleistocene–Holocene transition in Chilean Northern Patagonia

**DOI:** 10.3897/zookeys.1043.61706

**Published:** 2021-06-15

**Authors:** Francisco Tello, José R. Verdú, Michele Rossini, Mario Zunino

**Affiliations:** 1 Transdisciplinary Center for Quaternary Research (TAQUACH), Universidad Austral de Chile, Valdivia, Chile Universidad Austral de Chile Valdivia Chile; 2 I.U.I. CIBIO, Universidad de Alicante, Alicante, Spain Universidad de Alicante Alicante Spain; 3 Finnish Museum of Natural History (LUOMUS), University of Helsinki, Pohjoinen Rautatiekatu 13, Helsinki, 00014, Finland University of Helsinki Helsinki Finland; 4 Scuola di Biodiversità, Polo universitario Asti Studi Superiori, Asti, Italy Polo universitario Asti Studi Superiori Asti Italy

**Keywords:** Dung beetle, extinction, fossil beetles, new species, Pleistocene, South America

## Abstract

The South American Pleistocene–Holocene transition has been characterized by drastic climatic and diversity changes. These rapid changes induced one of the largest and most recent extinctions in the megafauna at the continental scale. However, examples of the extinction of small animals (e.g., insects) are scarce, and the underlying causes of the extinction have been little studied. In this work, a new extinct dung beetle species is described from a late Pleistocene sequence (~15.2 k cal yr BP) at the paleoarcheological site Pilauco, Chilean Northern Patagonia. Based on morphological characters, this fossil is considered to belong to the genus *Onthophagus* Latreille, 1802 and named *Onthophagus pilauco***sp. nov.** We carried out a comprehensive revision of related groups, and we analyzed the possible mechanism of diversification and extinction of this new species. We hypothesize that *Onthophagus pilauco***sp. nov.** diversified as a member of the *osculatii* species-complex following migration processes related to the Great American Biotic Interchange (~3 Ma). The extinction of *O. pilauco***sp. nov.** may be related to massive defaunation and climatic changes recorded in the Plesitocene-Holocene transition (12.8 k cal yr BP). This finding is the first record of this genus in Chile, and provides new evidence to support the collateral-extinction hypothesis related to the defaunation.

## Introduction

The South American Pleistocene–Holocene transition (~16.0–11.0 k cal yr BP) has been characterized by drastic changes in climatic conditions, animal and plant diversity, and types of early-human occupation (Dillehay 1989; Borrero et al. 1998; Dillehay et al. 2015). There is currently no clear paleontological consensus concerning the mechanisms that facilitated these processes and multiple hypotheses seem to provide equally robust explanations for these paleoecological events, which include transformations induced by climatic and anthropogenic factors. The latest hypothesis is based on stochastic changes induced by cosmic impact (i.e., the Younger Dryas bolide-impact hypothesis, ~12.8 k cal yr BP), which resulted in large fires that contributed to a rapid overturn in species, and climatic and environmental conditions in both hemispheres (Firestone et al. 2007). This assumption is supported by evidence of extraterrestrial material and charcoal spherules, which have been found at several paleontological sites distributed across four continents (e.g., Pino et al. 2019; Wolbach et al. 2020).

In addition, the extinction of megafauna caused drastic changes in forest ecosystems, as these animals (e.g., the Gomphotheriidae) were fundamental in the past to support a series of important trophic relations (Owen-Smith 1987; Barnosky et al. 2016; González-Guarda et al. 2017). Thus, megafauna-species-dependent (e.g., parasitic insects and dung beetles) are very likely to have suffered the loss of these large animals. As a consequence, these organisms likely experienced major changes in their community compositions, along with the extinction of many species (Zinovyev 2011; Ashworth and Nelson 2014; Tello et al. 2017). As an example, *Cobboldia russanovi* Grunin, 1973 (Gasterophilidae) was a mammoth-botfly that became extinct at the end of the Pleistocene in Russia due to the loss of its host (Kuzmina and Korotyaev 2019, and references therein).

In South America, most of the evidence of the extinction of dung beetle fauna is based on fossil breeding balls (i.e., ichnospecies) in the early, middle and late Pleistocene (Cantil et al. 2013, Sánchez et al. 2013) and the unique dung beetle fossil remains recovered from Coprinisphaeridae inchnofossils (Zunino 2013). To date, examples of changes in fossil insect assemblages and the collateral extinction of insect species caused by the loss of large mammals in the Pleistocene–Holocene transition are still scarce.

In this study, we analyzed fossil remains from a late Pleistocene sequence collected at the paleoarcheological site Pilauco in Chilean Northern Patagonia. The fossil remains are tentatively classified in the *Onthophagus osculatii* species-complex (Coleoptera: Scarabaeidae: Scarabaeinae) (Rossini et al. 2018a). The extant dung beetle genus *Onthophagus* Latreille, 1802 comprises about 200 valid species in the New World. This number is greatly overshadowed by the impressive diversity of the genus in the Afrotropical, Oriental, and Palearctic regions, which are home to over 1000, 600 and 400 species, respectively. In recent years, scientific expeditions and studies on biodiversity carried out in remote and still unexplored areas of the American continent, have made it possible to obtain a great deal of information about the natural history of certain specimens, alongside the description of an increasing number of new species. However, the genus *Onthophagus* has never been recorded from Chile (Elgueta 2000; González-Chang and Pinochet 2015). Furthermore, the small number of fossil *Onthophagus* species described so far have been found in Europe, in sites dated from the middle to upper Paleocene (61.6–56 Ma) to middle Miocene (14–13.5 Ma), and a more recent North American fossil dated from the upper Pleistocene (0.068–0.004 Ma) (Tarasov et al. 2016).

The main goals of this study were to analyze the morphology of the fossil remains, to discuss the paleoecological implications and biogeographical distributions of the extant Chilean dung beetle fauna, with emphasis on South American *Onthophagus*, and to suggest a taxonomic placement for these fossil remains.

## Study site and paleontological context

**Figure 1.**
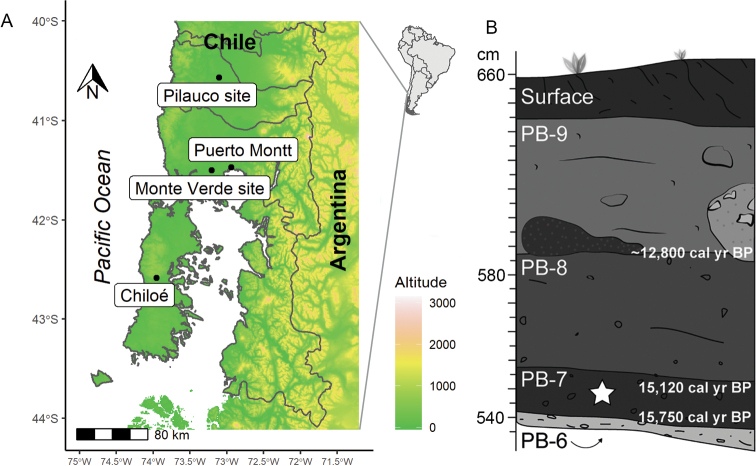
Type locality of *Onthophagus pilauco* sp. nov. **A** shaded elevation map shows Pilauco and Monte Verde sites. Representation of profile of a grid of the Pilauco site **B** symbol (star) indicates the layer in which *Onthophagus pilauco* sp. nov. was collected.

The Pilauco archeological and paleontological site is located in the city of Osorno, Chilean Northern Patagonia (40°34'S, 73°07'W) (Fig. 1A). Pilauco is considered to be a well-developed site at which to study the Pleistocene–Holocene transition due to its very obvious stratigraphy, large amount of animal and plant fossil remains, evidence of early-human occupation, and sedimentary record of the Younger Dryas bolide-impact at 12.8k cal yr BP (Pino et al. 2013, 2019, Moreno et al. 2019, Navarro-Harris et al. 2019). The current weather regime in Osorno (11.4 °C mean annual temperature, ~1300 mm precipitation per year) indicates temperate-warm climatic conditions.

### Stratigraphy and age

Stratigraphically, the Pilauco site is composed of four principal beds (Fig. 1B) that were deposited in fluvial, colluvial and palustrine environments (Pino et al. 2019). The basal bed (PB-6) is an unconsolidated sandy conglomerate, containing abundant, well-rounded pebbles and boulders. The overlying bed (PB-7) contains most of the extinct megafaunal remains, and is composed of an organic-rich sand with isolated colluvium-derived pebbles. Bed PB-8 is very similar to PB-7, although it contains lower abundances of mammal fossils and lithic artifacts. The Younger Dryas bolide-impact layer can be seen at the interface between beds PB-8 and PB-9, with PB-9 recording major changes in environmental conditions (Pino et al. 2019, 2020). Pino et al. (2019) proposed a Bayesian age model based on 36 accelerator mass spectrometry radiocarbon dates. These dates were calibrated according to the Southern Hemispheric calibration curve (SHIntCal13), providing an age range for Pilauco of between 16,400 and 4340 cal yr BP. According to the age model, the age of the fossil beetle is 15,200 cal yr BP (Fig. 1B), corresponding to the PB-7 bed.

### Paleoclimatic, paleoenvironmental and paleofaunistic records

Pollen records have recently indicated that the environment associated with bed PB-7 contained mainly non-arboreal taxa, such as Poaceae, Asteraceae, Solanaceae, and an aquatic flora (Abarzúa et al. 2020). Additionally, arboreal species, such as *Saxegothaea conspicua* Lindl., *Nothofagus dombeyi*-type and *Weinmannia trichosperma* Cav., were present in lesser proportions in the palynological record (Abarzúa et al. 2020). These proxies suggest that the climatic conditions were cold (at least ~4 °C lower) and humid compared to the current climate at the same locality. The presence of slightly arboreal temperate species of modern Chilean Northern Patagonia here between 16.0 and 14.0 k cal yr BP indicates very humid and cold climatic conditions. Additionally, the megafaunal bones at Pilauco correspond to several extinct taxa, including cf. *Notiomastodon platensis* (Ameghino, 1888) (Gomphotheriidae), Equus (Amerhippus) andium (Branco, 1883) (Equidae), *Xenarthra* sp. and cf. *Hemiauchenia paradoxa* (Gervais & Ameghino, 1880) (Camelidae), with the most abundant remains belonging to gomphotherids (Recabarren et al. 2011, Recabarren 2020). As for fossilized beetles, 22 species, belonging to 14 families, have been recorded from the Pilauco site. Among these, Curculionidae, Carabidae, Staphylinidae, Hydrophilidae and Scarabaeidae are the most abundant families (Tello et al. 2017, 2019). According to recent paleo-inferences based on beetle records, Pilauco was dominated by large mammals and climatic transition. When compared with modern beetle assemblages, there was a persistent rich beetle fauna, which included dung beetles and other coprophilous insects (Tello et al. 2017, 2019; Tello and Torres 2020).

## Materials and methods

### Abbreviations

**CEMT** Seção de Entomologia da Coleção Zoológica, Universidade Federal de Mato, Grosso, Cuiabá, Brazil;

**CMNC**Canadian Museum of Nature, Gatineau, Quebec, Canada;

**MPDO** Museo Pleistocénico de Osorno, Osorno, Chile;

**MZ** Mario Zunino private collection, Asti, Italy;

**MZUF**Museo di Storia Naturale dell’Università di Firenze, Florence, Italy;

**NMPC**Národní Muzeum, Prague, Czech Republic.

### Drawings and determination of fossil remains

**Figure 2.**
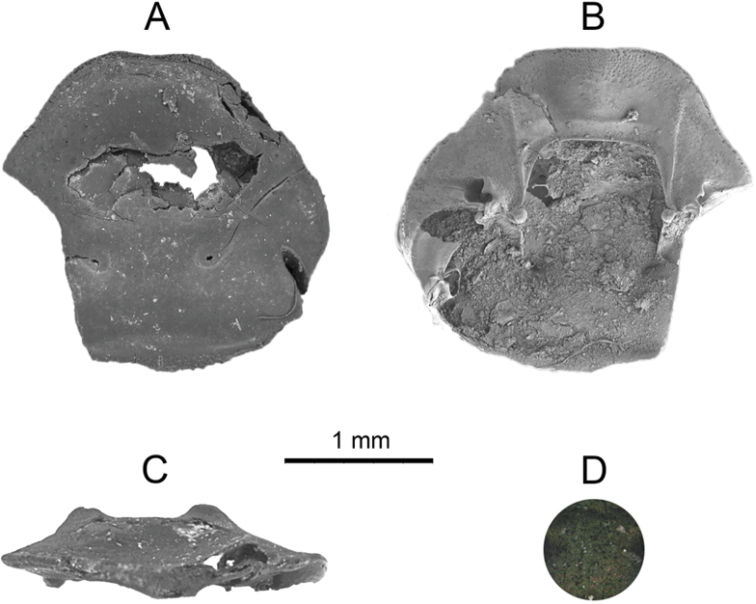
Holotype of *Onthophagus pilauco* sp. nov. **A** dorsal **B** ventral and **C** frontal views **D** detail of the microsculpture.

The fossil beetle remains were recovered from the sediment using an adaptation of the water flotation technique described by Hoganson et al. (1989) (see also Tello and Torres 2020). The remains were collected from grid 18AC, at an elevation of 384 cm in bed PB-7. The age span of this bed is ~16.0 to 14.0 k cal yr BP (Pino et al. 2020). The taxonomic placement suggested for the fossil was made after detailed examination and a comparison with multiple modern specimens of South American *Onthophagus* species deposited in the CEMT, CMNC, MZUF, MZ and NMPC collections. For the taxonomic nomenclature, we followed Rossini et al. (2018a, b). Figure 1A was obtained using R software v4.0.3. Figure 2A, 2B was obtained using a scanning electron microscope (variable pressure, EVO MC10), and Fig. 2C, D was obtained using a Leica M205C camera. All figures were processed using Adobe Photoshop 2019 CC. Type material is deposited in the MPDO insect collection, Osorno, Chile.

## Results

### Systematic paleontology

#### Order Coleoptera Linnaeus, 1758

**Suborder Polyphaga Emery, 1886**

**Family Scarabaeidae Latreille, 1802**

##### Genus *Onthophagus* Latreille, 1802

###### Type species Onthophagus pilauco sp. nov.

Taxon classificationAnimaliaColeopteraScarabaeidae

9E338F72-5BB1-5818-9366-24A7AFB05F2B

http://zoobank.org/9B203D54-E27A-432D-8AC8-ED7C33ED2F83

Fig. 2

####### Description.

***Holotype*. Male**, minor form. Clypeus sub-trapezoidal and slightly elongated forward, with anterior margin narrowly and slightly reflexed, head margin barely sinuated at the clypeo-genal junction. Fronto-clypeal region without carina, frons with two close, weak tubercles, strongly advanced in position, in line with the anterior margin of the eyes (Fig. 2A, C). Head surface very finely and evenly punctate. Latero-clypeal region with deeper ocellate punctures. Color dark with metallic green to bronze sheen (Fig. 2D). Pronotum and elytra not found.

**Female** unknown.

####### Diagnosis.

*Onthophagus pilauco* sp. nov. is considered to be a close relative of *O. confusus* Boucomont, 1932 and *O. insularis* Boheman, 1858, as it shares the following morphological characters with these species: sub-trapezoidal shape of the clypeus; small, slightly deeper cephalic punctation, coupled with very shallow wrinkles in proximity to the genal and clypeal margins. Although the fronto-clypeal region is significantly damaged, there is no indication of a possible carina.

####### Proposed English and Spanish vernacular names.

The Pilauco dung beetle (EN) and estercolero de Pilauco (ES).

####### Etymology.

The name of the new species refers to the archeopaleontological site from which the fossil remains were collected.

## Discussion

From our observations, the fossil remains found at Pilauco correspond to a new and extinct species of the genus *Onthophagus*, closely related to the *hircus* group. *Onthophagus pilauco* sp. nov. represents the first record of an endemic species of this genus in Chile. Moreover, this record brings new evidence of beetle extinction related to the Pleistocene–Holocene transition and massive defaunation after a possible cosmic impact and/or YD cooling reversal events.

### Morphological delimitation of the fossil record

Despite the beetle remains only being represented by cephalic parts (clypeus, right gena and frons; fronto-clypeal region partly damaged; left gena absent; see Fig. 2A–D), it is clear that they belong to the genus *Onthophagus.* Close scrutiny of the fossil remains, along with an extensive analysis of multiple specimens belonging to extant American *Onthophagus* led us to assign *O. pilauco* to the *O. hircus* group, and more precisely to the *osculatii* species-complex (Rossini et al. 2018a). The two cephalic horn-like tubercles may indicate that the remains belong to a male specimen, probably a minor form. The physical location of the cephalic tubercles is rather unique in the modern American *Onthophagus* fauna. They rise in a very advanced position, in line with the anterior margin of the eyes, and are quite close to each other. The combination of these two characteristics has only ever been found in an undescribed *Onthophagus* from Costa Rica, which was included in the same species group, but in a different taxonomic complex (Rossini, pers. comm. 2020). Close cephalic tubercles are also found in female specimens of species belonging to the *Onthophagus clypeatus, dicranius* and *mirabilis* groups, but they are always situated at the front, and never as advanced as in *O. pilauco*. Also, the shape of the clypeus in these females is always triangular or evenly curved, terminating at the apex with a margin slightly to distinctly emarginated (with two obtuse teeth).

### Hypothesis for the speciation and extinction of *Onthophagus pilauco*

Comprehensive knowledge of the Chilean beetle fauna suggests that only a few species (nine) can be considered to be exclusively associated with dung resources (González-Chang and Pinochet 2015). Thus, six species have been assigned to the Scarabaeidae family (excluding the saprophagous Aphodiinae species): two species belonging to Deltochilini tribe: *Megathopa villosa* Escholtz and *Scybalophagus rugosus* (Blanchard); and four species with uncertain taxonomic position (*incertae sedis sensu*Tarasov and Dimitrov 2016): *Tesserodoniella elguetai* Vaz-de Mello & Halffter, *T. meridionalis* Vaz-de Mello & Halffter, *Homocopris punctatissimus* (Curtis), and *H. torulosus* (Escholtz) (González-Chang and Pinochet 2015). The placement of the genus *Homocopris* in a suprageneric group requires further research. Additionally, three dung beetle species belong to the family Geotrupidae Latreille, 1802, represented in Chile by the subfamily Taurocerastinae: *Frickius costulatus* Germain, *F. variolosus* Germain and *Taurocerastes patagonicus*, which are distributed across central and southern Chile.

**Figure 3.**
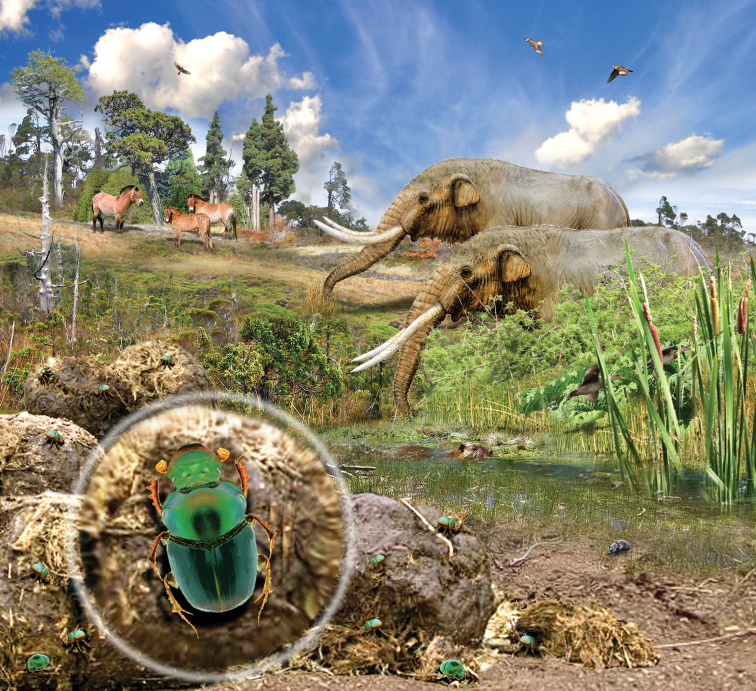
Artistic reconstruction of *Onthophagus pilauco* sp. nov. and its palaeoenvironment (by Mauricio Alvarez).

The Deltochilini group is distributed in the pantropical zone, being especially diversified in the northern areas of South America. The genus *Tesserodoniella* is related to the Australian genera *Tesserodon* and *Aptenocanthon* and their ancestors probably originated from the Gondwana supercontinent (Vaz-De-Mello and Halffter 2006). In contrast, the extant American *Onthophagus* is a result of migrations to the continent by intercontinental connections, the current remnants of which are known as Beringia components. These migratory processes occurred at different times and under different geographical and climatic conditions, and involved different ancestors, all belonging to the subgenus Onthophagus sensu stricto (Rossini et al. 2018a, b, Halffter et al. 2019, Zunino and Halffter 2019). Additionally, after the definitive closure of the Isthmus of Panama (~3 Ma), which eliminated natural barriers, Central America became a permanent bridge from one continent to another, improving migratory conditions for large animals and dung beetles. Therefore, we suggest that the ancestors of *O. pilauco* migrated to South America during the Great American Biotic Interchange, following large mammals at the end of the Pliocene (3 Ma). This migratory mechanism has been suggested for the extant related *hircus* group, which arrived by crossing the Andes via the Huancabamba Depression, similarly to other extinct dung beetles (e.g., *Phanaeus violetae* Zunino, 2013). Intra-continental migratory patterns have also been reported in extant species of dung beetles that have rapidly colonized new habitats following cattle migrations (e.g., *Digitonthophagus gazella* (Fabricius, 1787); see Noriega et al. 2020). Thus, ancestors of the *osculatii* species-complex diverged in situ in Chilean areas, resulting in the evolution of *O. pilauco* (Fig. 3). This speciation hypothesis is supported by several studies on Pleistocene Patagonian landscapes (~180 ka and 26 ka) that have suggested that the rapid contraction and expansion of ice cover has induced drastic changes in biotic distributions and prompted diversification in different groups of organisms (e.g., in amphibians: Nuñez et al. 2020; in mammals: Himes et al. 2008). Moreover, the presence of extant endemic species belonging to the *osculatii* species-complex in western Ecuador and northwestern Peru [*O. confusus* and *O. insularis* (Rossini et al. 2018a)] suggests that *O. pilauco* could be a species endemic to southern Chile.

On the other hand, the extinction of large animals in South American Pleistocene environments has invoked multiple climate- and human-activity-related hypotheses, and interactions between them (Barnosky et al. 2004). The causes of small animal (<60 kg) extinctions, and the implications of changes in species compositions and distributions, are poorly understood. Owen-Smith (1987) proposed the ‘keystone herbivore’ hypothesis, which provides a framework to explain the simultaneous extinctions of animals not obviously made extinct by the previous causes. Additionally, a possible cosmic impact may have generated the Younger Dryas cooling oscillation (12.8 and 11.0 k cal yr BP), resulting in a rapid defaunation process, including smaller taxa (Firestone et al. 2007; Pino et al. 2019; Wolbach et al. 2020). In this case, we suggest that both the rapid climatic changes and the extensive defaunation in South America could be determining factors in the extinction of *O. pilauco* during the Pleistocene–Holocene transition.

## Supplementary Material

XML Treatment for Onthophagus pilauco
